# Separation Properties of Wastewater Containing O/W Emulsion Using Ceramic Microfiltration/Ultrafiltration (MF/UF) Membranes

**DOI:** 10.3390/membranes3020087

**Published:** 2013-06-21

**Authors:** Kazuho Nakamura, Kanji Matsumoto

**Affiliations:** Division of Materials Science and Chemical Engineering, Graduate School of Engineering, Yokohama National University, 79-5 Tokiwadai Hodogaya-ku, Yokohama 240-8501, Japan; E-Mail: k-mtmt@ynu.ac.jp

**Keywords:** ultrafiltration, microfiltration, ceramic membrane, O/W emulsion, gel-polarization model

## Abstract

Washing systems using water soluble detergent are used in electrical and mechanical industries and the wastewater containing O/W emulsion are discharged from these systems. Membrane filtration has large potential for the efficient separation of O/W emulsion for reuses of treated water and detergent. The separation properties of O/W emulsions by cross-flow microfiltration and ultrafiltration were studied with ceramic MF and UF membranes. The effects of pore size; applied pressure; cross-flow velocity; and detergent concentration on rejection of O/W emulsion and flux were systematically studied. At the condition achieving complete separation of O/W emulsion the pressure-independent flux was observed and this flux behavior was explained by gel-polarization model. The O/W emulsion tended to permeate through the membrane at the conditions of larger pore size; higher emulsion concentration; and higher pressure. The O/W emulsion could permeate the membrane pore structure by destruction or deformation. These results imply the stability of O/W emulsion in the gel-layer formed on membrane surface play an important role in the separation properties. The O/W emulsion was concentrated by batch cross-flow concentration filtration and the flux decline during the concentration filtration was explained by the gel- polarization model.

## 1. Introduction

Washing systems using water soluble detergent are used in electrical and mechanical industries instead of the systems using organic solvent and the wastewater containing O/W emulsion is discharged from these systems. The methods of reducing the wastewater volume and recycling of the treated water and detergent are required. The microfiltration (MF) and ultrafiltration (UF) has large potential for the efficient separation and concentration of O/W emulsion [1,2]. The combined UF and reverse osmosis (RO) processes for waste O/W emulsion in the cable and wire industry showed the excellent performances for reuse of treated water [3]. The UF and nanofiltration (NF) process also showed good filtration performance in the treatment of metal-working fluids [4]. 

In the MF and UF of O/W emulsion the pressure independent flux was commonly observed and the flux behaviors were explained by pore clogging [5,6], concentration polarization [4,7] and/or gel-polarization [6,8] models. The flux can be improved by enhancing the mass-transfer at the surface of membrane [7,9,10]. The rejection of O/W emulsion depends on various experimental parameters including membrane pore size, membrane material, operating conditions, O/W emulsion concentration and stability, and coexistence materials in feed stream [11,12]. In general while the O/W emulsions can be rejected by UF membranes, they can pass through MF membranes. The phenomena at membrane surface during MF of O/W emulsions are quite complex because of the complexity of force applied to emulsions including surface tension between emulsion and pore surface, filtration pressure, and shear stress from feed fluid, *etc.* So it is difficult to predict the flux and the penetration properties of O/W emulsion through membrane pore structure. 

In this study the separation properties of O/W emulsions by cross-flow MF and UF were studied with ceramic MF and UF membranes. The effects of pore size, applied pressure, cross-flow velocity, and detergent concentration on rejection of O/W emulsion and flux were systematically studied. The flux behaviors were discussed by the gel-polarization model. The conditions where O/W emulsion can permeate the membranes were discussed in terms of the stability of O/W emulsion in the gel-layer.

## 2. Experimental Section

The UF and MF ceramic membranes used were in the form of φ 30 mm × 500 mm (φ 3 mm and 37 channels) monolith type (NGK Cefilt MF/UF series) supplied from NGK FILTECH Ltd. The effective filtration area was 0.17 m^2^. The nominal pore size of the MF membrane was 100 nm and those of the UF membranes were 5, 10, 50 nm. The corresponding molecular weight cut off (MWCO) of these UF membranes were 20, 50, and 150 kDa. MF membrane was made of alumina. UF membranes had a thin titania layer coated on the surface of supporting alumina MF membrane.

The detergent used was CLEANTHROUGH LC841 (KAO. Co., Ltd.), which is widely used in precision machine and liquid crystal panel industries. The composition of this detergent was hydrocarbon (60%), EO/PO nonionic surfactant (30%), and water (10%). Stable O/W emulsion suspension could be prepared by just diluting this detergent with water. The prepared detergent suspension contains both O/W emulsion and soluble surfactants. The detergent concentration in feed tank was adjusted to 0.5–3.0 wt %. The median size of O/W emulsion in the feed tank was about 2 µm. This suspension was mainly used as a model wastewater containing O/W emulsion. In some experiments machine oil (Showa Shell Sekiyu K.K., Speed C15) was added to the feed detergent suspension (1.0 wt %) in the concentration range 0.2–0.9 wt %. The size of O/W emulsion was measured with a laser scattering particle size analyzer (Seishin enterprise, LMS-24) and the measurement was done just after sampling. The quality of filtrate was checked with a total organic carbon (TOC) analyzer (Shimadzu, TOC-500).

**Figure 1:**
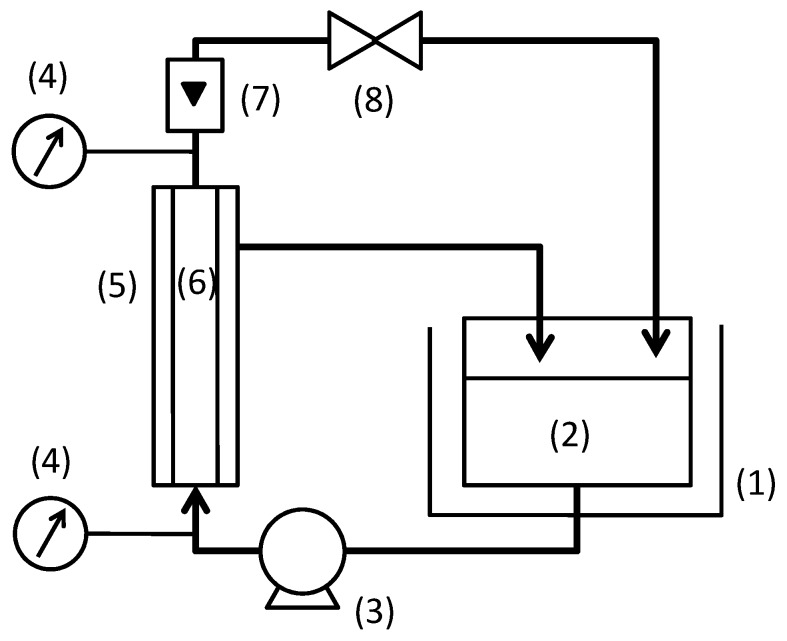
Experimental set-up for cross-flow filtration: (1) thermostatic bath; (2) feed tank; (3) pump; (4) pressure gauge; (5) membrane module; (6) ceramic membrane; (7) flow meter; (8) valve.

The experimental set-up is shown in Figure 1. The feed suspension was filtrated in the cross-flow filtration mode. Both total recycle filtration, in which the permeate was returned to feed tank, and batch concentration filtration were conducted. The batch concentration filtration was conducted until the volume concentration factor became 7. The temperature of solution was kept constant at 60 °C. Filtration pressure and cross-flow velocity were varied from 50 to 300 kPa and from 0.5 to 3.0 m/s, respectively. The flux became constant in a short time for any filtration conditions. Hence, the flux and rejection of TOC components were measured at fifteen minutes after changing filtration condition. The total filtration time for the measurements were about 6 h for one feed suspension condition in the total recycle filtration and about 2 to 10 h for a batch concentration filtration. 

## 3. Results and Discussion

### 3.1. Filtration Properties in Retentive Condition with UF Membrane (Nominal Pore Size 5 nm)

**Figure 2:**
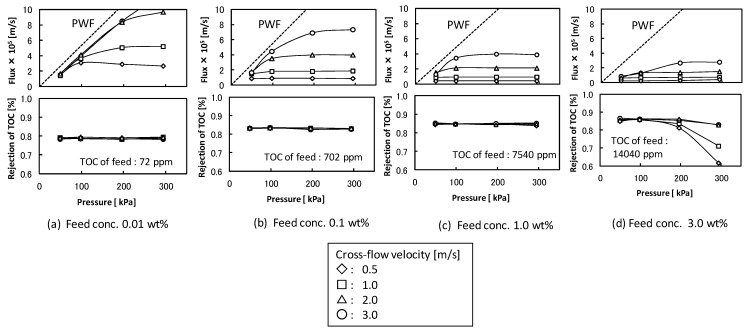
Effects of cross-flow velocity, filtration pressure, and feed concentration on the flux and the rejection of total organic carbon (TOC) components in ultrafiltration (UF) membrane (nominal pore size 5 nm). The dashed lines show pure water flux (PWF).

The effects of cross-flow velocity, filtration pressure, and feed concentration on the flux and rejection of TOC components in the total recycle filtration were studied with UF membrane (pore size 5 nm). Figure 2 shows the filtration properties. The flux increased with increasing filtration pressure at lower pressure and became almost constant regardless of filtration pressure at higher filtration pressure. The pressure-independent flux increased with increasing cross-flow velocity and decreased with increasing feed detergent concentration. The extent of the changes in pressure-independent flux in higher cross-flow velocity was larger than that in lower cross-flow velocity. These behaviors of flux were typically observed in UF of protein or colloid suspension [2]. The rejection of TOC component was about 0.8 and the permeate solution was transparent except for the condition in higher filtration pressure at the feed concentration of 3.0 wt %. In the conditions at the feed concentration under 1.0 wt % the O/W emulsion was fully rejected by the membrane and the TOC components in the permeate solution might be soluble surfactant. In the condition of the feed concentration of 3.0 wt % and the higher filtration pressure (Figure 2d) the permeate solution became turbid due to permeation of O/W emulsion. In this condition the O/W emulsions accumulated at the membrane surface might become unstable and permeate the membrane structure by destruction or deformation.

**Figure 3:**
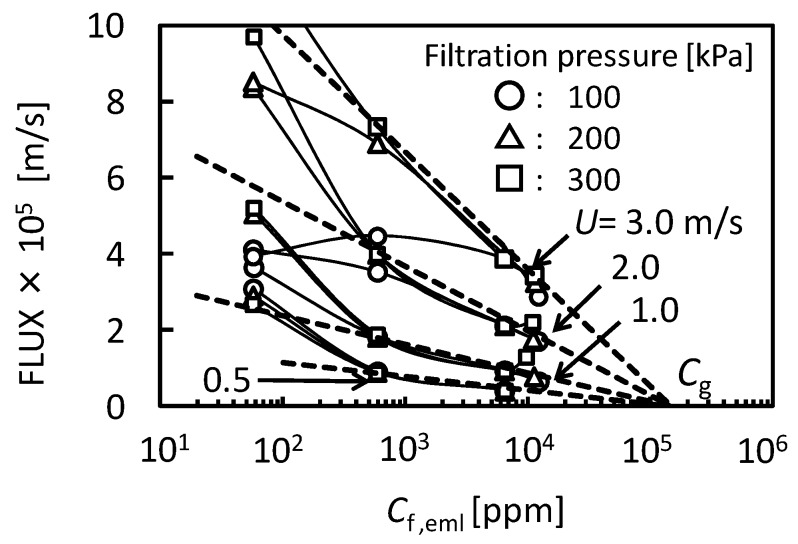
The relationship between the flux observed in UF (pore size 5 nm) membrane and O/W emulsion concentration as TOC. The dashed lines show linear regressions based on the gel-polarization model.

From these observations “Gel-Polarization” model can be applied to explain the flux behavior. In this model if the filtration pressure and the feed concentration are high enough, the accumulated O/W emulsions, which is the component of the detergent suspension rejected at the membrane surface, will form a gel-layer. The gel-layer can offer the major resistance to flow and will thicken or compact just compensating for the increased filtration pressure by an equal increase in the resistance. Therefore, flux will decrease to its original value in the steady state and is independent of pressure and is solely determined by the back-diffusive transport. For a fully retentive membrane the pressure-independent flux is given as:

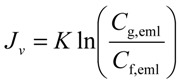
(1)
where *J_v_* is permeate flux, *K* is mass transfer coefficient, *C*_f,eml_ is O/W emulsion concentration in feed, and *C*_g,eml_ is O/W emulsion concentration in gel-layer formed on the membrane surface. Figure 3 shows the relationship between the flux observed in UF (nominal pore size 5 nm) membrane and O/W emulsion concentration as TOC. The flux clearly decreased with increasing *C*_f,eml_. In the higher concentration the flux depended on cross-flow velocity, *U*, regardless of filtration pressure. The dashed lines show linear regressions based on the gel-polarization model predicted by Equation (1). In the higher concentration the experimental plots are good agreement with the model predictions. Extrapolation of the data to zero filtrate flux roughly gives the value of *C*_g,eml_ = 1.5 × 10^5^ ppm regardless of cross-flow velocity. Mass transfer coefficient *K* can be determined from the slope of lines as follows:

at *U* = 0.5 m/s, *K* = 1.58 × 10^−6^ m/s
at *U* = 1.0 m/s, *K* = 3.25 × 10^−6^ m/s
at *U* = 2.0 m/s, *K* = 7.33 × 10^−6^ m/s
at *U* = 3.0 m/s, *K* = 1.34 × 10^−5^ m/s

From these data *K* can be expressed as a function of *U*.

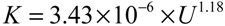
(2)
By using these determined parameters of *K* and *C*_g_ the pressure-independent flux observed in the fully retentive filtration condition was correlated to experimental parameters of *U* and *C*_f,eml_ with Equations (1) and (2). 

### 3.2. The Conditions of O/W Emulsion Permeate Through Membrane

In some filtration condition the O/W emulsions can permeate through membrane pore structure due to destruction or deformation of O/W emulsions even if UF membrane (nominal pore size 5 nm) is employed as shown in Figure 2d. Therefore the conditions where the O/W emulsions can permeate membrane were studied with membranes having a wide range of pore size and various filtration conditions in the total recycle filtration mode. 

**Figure 4:**
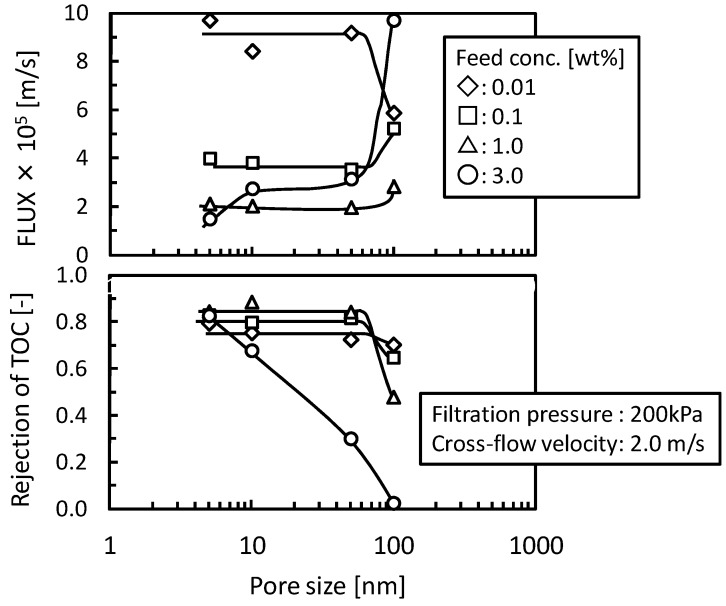
Effects of the feed concentration on flux and the rejection of TOC component of UF and microfiltration (MF) membranes as functions of nominal pore size.

The Figure 4 shows the effects of feed concentration and pore size of UF and MF membranes on flux and the rejection of TOC components. In UF membranes (nominal pore size, 5, 10, and 50 nm) the O/W emulsion was fully rejected at below the feed concentration of 1.0 wt % and the flux showed almost the same value depending feed concentration regardless of pore size. In these conditions the filtration properties would substantially depend on the gel-layer formed on the surface of membrane rather than membrane pore structure. In the condition of the feed concentration of 3.0 wt % the rejection of TOC components decreased with increasing pore size. The O/W emulsions might permeate the membrane structure by destruction or deformation in this concentration and the membrane having larger pore size tended to induce this permeation. In the MF membrane (nominal pore size 100 nm) the rejection of TOC components decreased reflecting O/W emulsion permeation and the flux showed different trends compared with the UF membranes. These results show that when the permeation of O/W emulsion occurred the stable gel-layer could not be formed on the surface of membrane and the gel-polarization model cannot be applied. 

**Figure 5:**
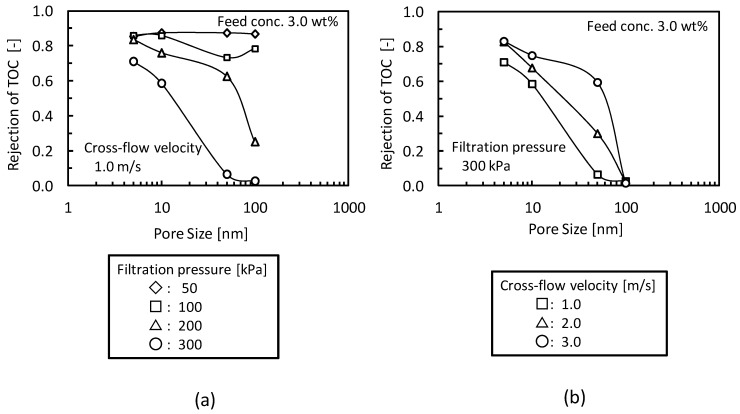
Effects of (**a**) filtration pressure and (**b**) cross-flow velocity on the rejection of TOC components.

Figure 5 shows the effects of (a) filtration pressure and (b) cross-flow velocity on the rejection of TOC components. In Figure 5a, the rejection of TOC components decreased with increasing filtration pressure. At lower filtration pressure O/W emulsions were fully rejected even if the MF membrane (nominal pore size 100 nm) was employed. The higher filtration pressure would force the emulsions into pore structure by destruction or deformation. In Figure 5b, the rejection of TOC components decreased with decreasing cross-flow velocity. In the lower cross-flow velocity condition the lower shear rates would promote accumulation of O/W emulsions, which will form gel-layer, on the membrane surface. The local environment of the higher O/W emulsion concentration in the gel-layer will make the O/W emulsions in the gel-layer more unstable. In the higher cross-velocity condition, the higher shear stress at the membrane surface will disturb the accumulation of O/W emulsions and lower the local concentration of O/W emulsions at membrane surface. According to the gel-polarization model both higher filtration pressure and lower cross-flow velocity, of which the conditions caused the permeation of O/W emulsion, will increase the gel-layer thickness. This observation shows that the concept of gel-layer formation could be useful not only for the analysis of the pressure-independent flux but also for the prediction of O/W emulsion permeation. 

**Figure 6:**
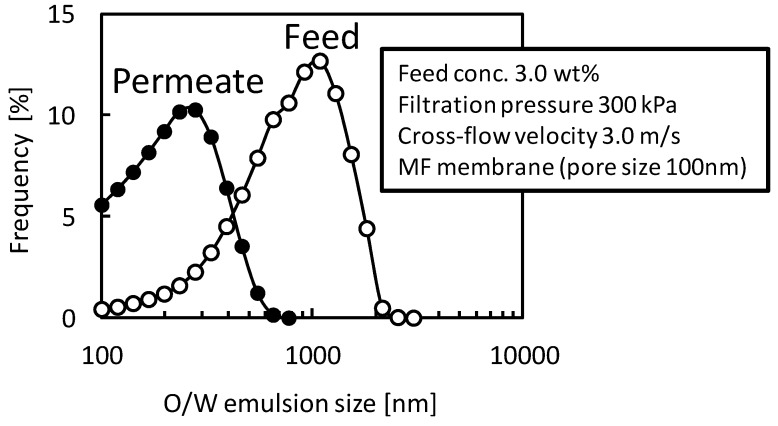
Size distributions of O/W emulsion in feed and permeate suspensions in the filtration with the MF membrane (nominal pore size 100 nm).

Figure 6 shows size distributions of O/W emulsion in feed and permeate suspension in a filtration with the MF membrane (nominal pore size 100 nm). In the filtration condition the rejection of O/W emulsion was almost zero. The size of O/W emulsion in the permeate suspension was clearly smaller than that in the feed suspension and was larger than membrane pore size of 100 nm. This observation supports that the O/W emulsion droplets will permeate the membrane structure due to destruction or deformation of droplets.

**Figure 7:**
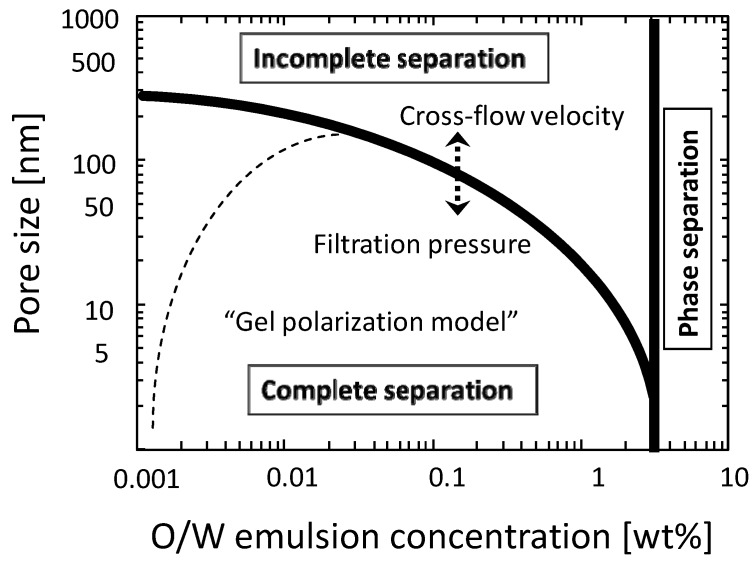
Diagram for the relationship between the membrane filtration properties and the filtration condition.

From these observations the relationship among membrane pore size, filtration condition, and the filtration properties is illustrated in Figure 7. This diagram can be divided into three regions which are the phase separation, the incomplete separation, and the complete separation regions. The liquid-liquid phase separation of the detergent suspension was observed in the detergent concentration of more than 3 wt %. In this region a pre-treatment process like gravity separation will be needed before membrane separation process. In the incomplete separation region the O/W emulsion can permeate membrane and in the complete separation region the O/W emulsion will be fully rejected. The line dividing these regions will depend on filtration pressure and cross-flow velocity. In the complete separation region the pressure-independent flux will be observed and explained by the gel-polarization model.

### 3.3. Addition of Machine Oil

The effect of machine oil (0.2–0.9 wt %) adding to the feed detergent suspension (1.0 wt %) on the filtration performance was studied with UF membrane (nominal pore size 5 nm). The rejection of TOC components and flux were hardly affected by the oil adding. The added oil was completely rejected. There was no effect on the filtration performance by adding the oil in the conditions studied. 

### 3.4. Batch Concentration Filtration with UF Membrane (Pore Size 5 nm)

**Figure 8:**
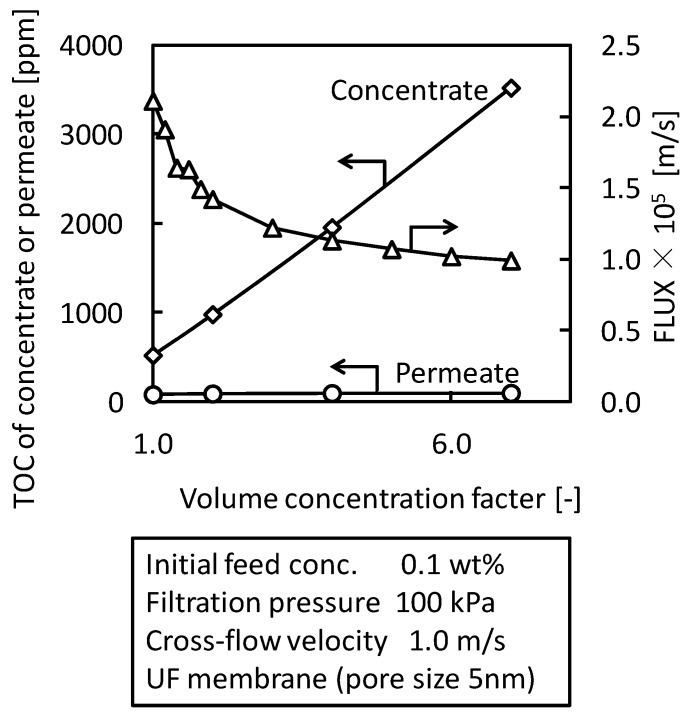
Concentration properties of O/W emulsion in batch cross-flow concentration filtration with UF membrane (pore size 5 nm).

The concentration properties of O/W emulsion were studied by batch cross-flow concentration filtration with UF membrane (nominal pore size 5 nm). Figure 8 shows a typical result of the concentration properties. TOC of the concentrate, which is an index of O/W emulsion concentration, increased in proportion to the volume concentration factor. TOC of the permeate was constant during the batch concentration filtration. These changes show that O/W emulsion was concentrated according to volume concentration factor and water soluble surfactant, which is the component of TOC in the permeate, was not concentrated by this concentration filtration. The flux gradually decreased with increasing the concentration factor during the concentration filtration. 

**Figure 9:**
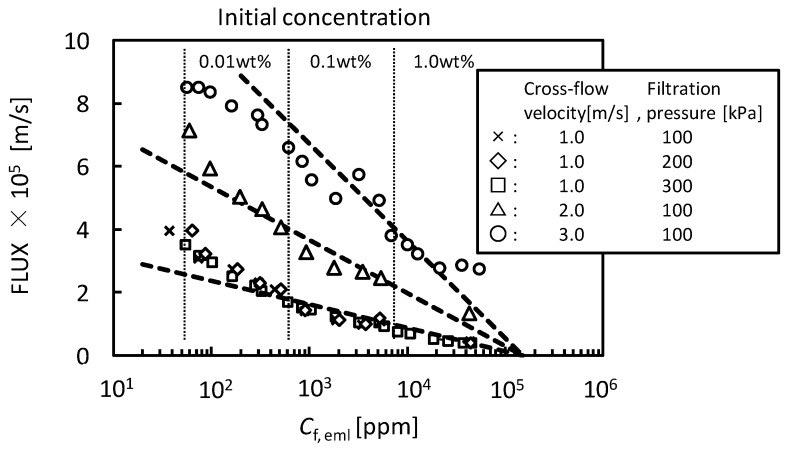
Concentration properties of O/W emulsion in batch cross-flow concentration filtration with UF membrane (nominal pore size 5 nm).

The behaviors of flux decline during the batch cross-flow concentration filtrations were analyzed by the gel-polarization model. Figure 9 shows the effects of filtration pressure, cross-flow velocity and initial feed concentration on flux. The flux decreased with increasing concentration of O/W emulsion during each concentration filtration. The filtration pressure had no effect on the behavior of flux while the flux increased with increasing cross-flow velocity. The dashed lines in Figure 9 show the flux predicted by the gel-polarization model using Equations (1) and (2) with parameters obtained above. The flux decline during the cross-flow concentration filtration followed these predicted lines. It is shown that the flux decline during the concentration filtration will be predicted by the gel-polarization model.

## 4. Conclusions

The separation and concentration properties of O/W emulsions by cross-flow microfiltration and ultrafiltration were studied with ceramic MF and UF membranes. The filtration condition where O/W emulsion can be fully rejected was clarified. The relationship among the membrane pore size, filtration condition (filtration pressure, cross-flow velocity, and feed concentration), and the filtration properties (rejection and flux) was summarized in a diagram (Figure 7). In the condition achieving complete separation of O/W emulsion the pressure-independent flux was observed and this flux behavior was explained by gel-polarization model. The gel-polarization model was also useful to explain the flux decline properties during cross-flow concentration filtration. 
